# Prevalence of IMPG1 and IMPG2 Mutations Leading to Retinitis Pigmentosa or Vitelliform Macular Dystrophy in a Cohort of Patients with Inherited Retinal Dystrophies

**DOI:** 10.3390/genes16010043

**Published:** 2025-01-01

**Authors:** Ming Yuan, Souradip Chatterjee, Monique Leys, J. Vernon Odom, Ezequiel M. Salido

**Affiliations:** 1 West Virginia School of Osteopathic Medicine, Lewisburg, WV 24901, USA; myuan@osteo.wvsom.edu; 2 Department of Biochemistry and Molecular Medicine, West Virginia University, Morgantown, WV 26506, USA; souradip.chatterjee@hsc.wvu.edu; 3 Department of Ophthalmology and Visual Sciences, West Virginia University, Morgantown, WV 26506, USA; monique.leys@hsc.wvu.edu (M.L.); odomj@wvumedicine.org (J.V.O.)

**Keywords:** interphotoreceptor matrix, IMPG1, IMPG2, retinitis pigmentosa, vitelliform macular dystrophy, IRDs

## Abstract

Background/Objectives: The interphotoreceptor matrix proteoglycans 1 and 2 (IMPG1 and IMPG2) are two interdependent proteoglycans of the interphotoreceptor matrix (IPM). Mutations in IMPG1 or IMPG2 are linked to retinal diseases such as retinitis pigmentosa (RP) and vitelliform macular dystrophy (VMD), yet the specific mutations responsible for each condition remain undefined. This study identifies mutations in IMPG1 and IMPG2 linked to either RP or VMD. It also provides an in-depth in silico analysis of these mutations’ structural and functional impact on protein domains, alongside a detailed examination of the corresponding disease phenotypes. Methods: From a cohort of 480 patients with inherited retinal diseases (IRDs), we identified seven patients with mutations in IMPG1 or IMPG2. Multimodal imaging was performed to assess the clinical phenotypes, including fundus photography, fundus autofluorescence, fluorescein angiography, and spectral domain optical coherence tomography (SD-OCT). We provide structure modeling and analysis of each variant. Results: Our findings indicate a prevalence of 1.45% of IRD patients being affected by IMPG mutations; two were diagnosed with RP and five with VMD. One VMD patient carried a novel IMPG1 p.Asp423Glu mutation. Most patients exhibited heterozygous mutations, and one RP patient presented a compound heterozygous mutation in IMPG2. Conclusions: This work describes a novel mutation and expands our understanding of the specific IMPG protein domains implicated in RP and VMD. Furthermore, it establishes, for the first time, the prevalence of IMPG mutations in an IRD population.

## 1. Introduction

The interphotoreceptor matrix (IPM) is a unique type of extracellular matrix (ECM) that envelops the inner and outer segments of photoreceptor cells [1]. It also functions as an interphase between the photoreceptors and the retinal pigment epithelium (RPE). The IPM plays several essential roles in the proper functioning and maintenance of the retina, such as providing structural support to photoreceptors [2] and facilitating the exchange of nutrients and metabolic waste between the photoreceptors and the RPE, including the transport of retinoids [3,4,5]. Understanding the primary roles of the IPM in retinal health and disease is crucial for developing targeted therapies for retinal degenerative disorders.

**Figure 1:**
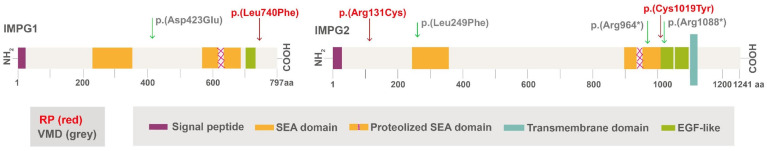
Scheme representation of IMPG1 and IMPG2 protein domains and found mutations leading to RP or VMD. The RP mutations are represented in red, while the VMD mutations are shown in grey. Two of our patients possess Arg964* mutation. Patient M83 with RP showed Cys1019Tyr in one allele and Arg131Cys in the other allele.

The interphotoreceptor matrix proteoglycans 1 and 2 (IMPG1 and IMPG2, IMPG1/2) are proteoglycans involved in the structural organization and maintenance of the IPM [6,7,8,9]. These proteoglycans, also known as SPACR and SPACRCAN or IPM150 and IPM200, are synthesized exclusively by photoreceptors and are highly conserved across various vertebrate species [6,9,10,11,12]. Both extracellular proteoglycans contain a signal peptide and possess multiple O- and N-glycosylation sites, which result in significant glycosylation and attachment of chondroitin sulfate chains (Figure 1) [10,13,14,15]. IMPG1/2 also possesses two SEA (Sperm protein, Enterokinase, and Agrin) domains, termed SEA-1 and SEA-2, based on their position in the polypeptide chain (Figure 1) [9,12]. Our earlier studies have shown that proteolytic cleavage of the SEA-2 domain is a normal part of IMPG1/2 maturation [16].

Inherited retinal diseases (IRDs) represent a group of genetically diverse conditions that cause progressive vision loss. Depending on the specific genetic mutations involved, these diseases can manifest in different forms [17,18]. Advances in molecular genetics have greatly improved our ability to diagnose and classify IRDs, but understanding the precise molecular mechanisms behind these conditions remains a significant challenge [19,20].

Mutations in the genes encoding IMPG1/2 are linked to two distinct diseases: retinitis pigmentosa (RP) [21,22,23,24,25] and vitelliform macular dystrophy (VMD) [26,27,28]. Prior research also linked a splice site mutation in *IMPG2* to Stargardt-like macular dystrophy disease, suggesting that mutations in *IMPG1/2* can lead to a wide spectrum of IRDs [29]. Despite the critical roles of *IMPG1/2* in visual function and their link to blinding conditions, the underlying mechanisms driving their pathogenesis remain largely unclear. This study provides new insights into the genotypic and phenotypic characteristics associated with IMPG1/2 mutations and explores the prevalence of these rare variants in IRDs.

RP is a heterogeneous group of genetic degenerative diseases and accounts for half of the IRDs [19]. Patients report night blindness and gradual peripheral vision loss due to dysfunctional rod photoreceptors [17]. As the disease progresses, the cones deteriorate, progressing to rod-cone dystrophy that can either be non-syndromic or syndromic/systemic [30]. A classic RP fundus view would show waxy optic discs, attenuated blood vessels, and bone spicules [17].

VMD is commonly linked to the *BEST1* and *PRPH2* genes [31,32,33]. However, in patients without those gene mutations, *IMPG1/IMPG2* gene mutations have been implicated [26,27,34]. In patients with VMD, lipofuscin material gathers under the macula, forming a vitelliform-like lesion in the subretinal space [35]. Acquired vitelliform lesions are frequently found in age-related macular degeneration (AMD) associated with drusen [36].

Despite RP and VMD being linked to mutations in *IMPG* genes, the mechanisms dictating whether a mutation leads to RP or VMD remain unclear. This study aims to clarify the genetic and phenotypic variability between these two diseases, specifically through identifying and analyzing novel mutations in *IMPG1/IMPG2* and their affected domains.

## 2. Methods

### 2.1. Subjects and Examinations

This study included 480 patients examined at the IRDs Clinic at the Department of Ophthalmology and Visual Sciences, West Virginia University, between March 2015 and June 2024. Inclusion criteria were based on clinical evaluation, family history, or prior diagnostic tests. All patients underwent genetic testing through next-generation sequencing (NGS) panels conducted by various commercial and research laboratories. All seven patients described in this study were sponsored by INVITAE (San Francisco, CA, US), which screened 248–330 genes associated with retinal dystrophies, including *IMPG1* and *IMPG2*.

Genetic testing and data collection were conducted in accordance with the principles outlined in the Declaration of Helsinki, with informed consent obtained from each participant. Electronic medical records were reviewed to gather clinical histories. Best corrected visual acuity (BCVA) was measured using Snellen acuity charts, and detailed retinal imaging was performed to assess structural and functional changes. Color fundus photography (FF) and fundus autofluorescence (FAF) imaging were carried out using a California Optos imaging system (Opthos, Dunfermline, UK). These imaging modalities were employed to detect atrophic, pigmentary, and vascular changes in the retina.

In addition to FF and FAF imaging, fluorescein angiography (FA) and optical coherence tomography (OCT) were performed using the Spectralis imaging system (Heidelberg Engineering, Heidelberg, Germany) to evaluate vascular abnormalities and retinal dystrophy-specific features. OCT imaging focused on identifying key indicators in RP cases, including peripheral retinal thinning, ellipsoid zone loss, cystoid macular edema (CME), and RPE changes. In VMD cases, OCT was used to assess vitelliform lesions, RPE elevation, photoreceptor layer disruption, and potential fluid accumulation. Furthermore, patients with RP underwent Goldmann visual field (GVF) testing (Haag-Streit, Bern, Switzerland) to evaluate peripheral vision loss.

### 2.2. Pathogenicity Prediction

To assess the pathogenicity of the *IMPG1* and *IMPG2* gene variants, we used several online tools, including SIFT (http://sift.jcvi.org/) [37], PolyPhen-2 (http://genetics.bwh.harvard.edu/pph2/, accessed on 10 November 2024) [38], MutationTaster (https://www.mutationtaster.org/, accessed on 10 November 2024) [39], CADD (https://cadd.gs.washington.edu/, accessed on 10 November 2024) [40], and FATHMM (http://fathmm.biocompute.org.uk/, accessed on 10 November 2024) [41]. These tools were used to predict the functional impact and deleteriousness of the variants.

### 2.3. Modeling and Evaluation of Modeled Structures

Protein structures of both reference and mutant forms of IMPG1 and IMPG2 were initially predicted using the I-TASSER tool (http://zhanggroup.org/I-TASSER/, accessed on 15 November 2024) [42], which employs replica exchange Monte Carlo simulations and the iterative threading assembly refinement algorithm to reassemble structural fragments from threading templates. The final selected models were evaluated using the SAVES server (https://saves.mbi.ucla.edu/, accessed on 15 November 2024), ensuring that the structure with the highest confidence score and over 80% of residues in the favored regions of the Ramachandran plot was chosen for further analysis.

### 2.4. Protein Stability Prediction

The impact of mutations on the stability of IMPG1 and IMPG2 proteins was evaluated using several computational tools, including mCSM (Mutation Cutoff Scanning Matrix) http://biosig.unimelb.edu.au/mcsm/, accessed on 16 November 2024 [43], SDM (Site-directed Mutator) https://veena.medschl.cam.ac.uk/sdm2 accessed on 16 November 2024 [44], DUET (http://biosig.unimelb.edu.au/duet/ 16 November 2024) [45], iMutant (https://folding.biofold.org/i-mutant/i-mutant2.0.html, accessed on 16 November 2024) [46], and MuPRO (http://mupro.proteomics.ics.uci.edu/ 16 November 2024) [47]. These tools calculate the Gibbs free energy (ΔΔG) changes to predict whether the mutation stabilizes or destabilizes the protein. The ΔΔG values were interpreted as either stabilizing or destabilizing based on the scoring system provided by each tool. Negative ΔΔG values generally indicate a destabilizing effect, while positive values suggest a stabilizing effect.

### 2.5. Interatomic Analysis in Reference and Mutant Proteins

The interactions between amino acid residues in the reference and mutant forms of IMPG1 and IMPG2 proteins were analyzed using DynaMut (http://biosig.unimelb.edu.au/, accessed on 16 November 2024). This tool utilizes normal mode analysis to assess changes in the protein structure and predict the effects on interatomic interactions [48].

### 2.6. Physiochemical Analysis

The molecular weight, theoretical isoelectric point (pI), total number of atoms, instability index, aliphatic index, grand average of hydropathy (GRAVY), and the ProtParam tool (https://web.expasy.org/protscale/, accessed on 23 November 2024) from the ExPASy (Expert Protein Analysis System) server was utilized. ProtParam allows for the computation of various physical and chemical parameters for a given protein sequence, providing insights into its stability, solubility, and hydrophobicity. By comparing these characteristics between the reference and mutant forms of the IMPG1 protein, we can gain valuable insights into potential structural and functional changes that may influence protein behavior and pathogenicity.

## 3. Results

Single gene variants linked to IRDs affect approximately 1 in 3000 (+/−1000) individuals worldwide [18]. In our study, 7 out of 480 patients with IRDs (1.45%) were found to have mutations in *IMPG* genes. Of these, two patients (0.41%) were diagnosed with RP, while five patients (1.04%) exhibited characteristics of VMD. These data reflect the prevalence of these mutations within the cohort of patients diagnosed with IRDs at the West Virginia University Eye Institute over a period of 9 years and 3 months. All seven patients with IMPG mutations were Caucasian.

Our findings show all missense mutations for the RP phenotype with dominant inheritance in IMPG1 and recessive in IMPG2 (Figure 1, red text). On the other hand, all patients expressing VMD harvest dominant mutations, which can be either mis- or non-sense (Figure 1, grey text). All the patients are represented in Table 1.

### 3.1. RP Patients

**Figure 2:**
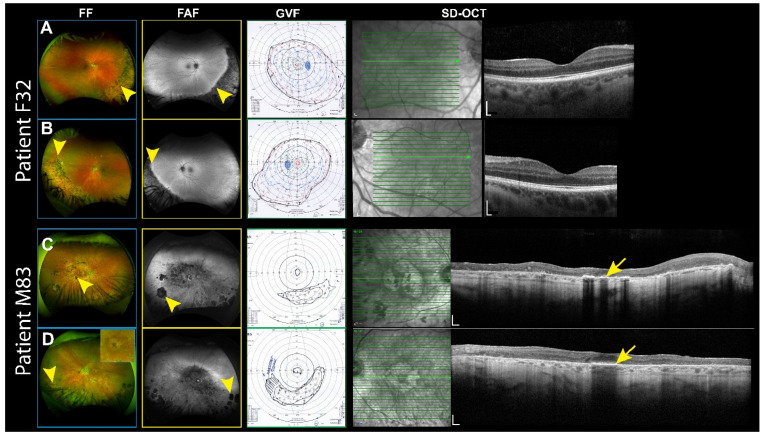
Multimodal imaging and functional assessment of RP in patients with IMPG1 and IMPG2 mutations. Right eye top, left eye bottom for each patient. (**A**,**B**) Patient F32, fundus photography (FF) reveals bone spicule pigmentation (yellow arrowhead). Fundus autofluorescence (FAF) imaging shows reduced autofluorescence (yellow arrowhead). SD-OCT shows standard foveal architecture with no significant retinal thinning. The Goldmann Visual field (GVF) shows superior depression. (**C**,**D**) Patient M83 FF reveals widespread atrophy (yellow arrows), with a bull’s eye pattern around the fovea (insert box). The FAF shows reduced autofluorescence (yellow arrowhead). The GVF test shows a central and inferior preserved field in both eyes. The SD-OCT shows partial conservation of the ellipsoid (yellow arrows).

Patient F32 is a 32-year-old female with a dominant missense mutation in the *IMPG1* gene (p.Leu740Phe), affecting a protein region near the EGF-like domain (Figure 1). Notably, previous studies have only described mutations impacting the proteolytic SEA domain in this region [49]. This finding suggests that the EGF-like domain has a role in the pathogenesis of the disease. Clinically, Patient F32 presented with sector RP primarily affecting the inferior retina (Figure 2A,B). She initially reported night vision problems to an optometrist, leading to a referral for further evaluation. The FF examination revealed dystrophy of the inferior retina, characterized by sectorial bone spicule pigmentation (yellow arrows). The FAF imaging showed reduced autofluorescence corresponding to a superior visual field defect in both eyes (yellow arrows). Despite these findings, her visual acuity and the macular ellipsoid zones were not notably compromised at the time of assessment. The best corrected visual acuity (BCVA) was 20/25 in both eyes.

The second RP patient is an 83-year-old male with two missense mutations in the *IMPG2* gene. He is heterozygous for the p.Cys1019Tyr mutation in one allele, which affects the EGF-1-like domain, and the p.Arg131Cys mutation in the other allele, which is located near the non-proteolytic SEA-1 domain. The Cys1019Tyr variant is a rare mutation found exclusively in the European (non-Finnish) population, with an estimated frequency of approximately 1 in 93,300 individuals. In contrast, the Arg131Cys variant is more common, occurring at a frequency of about 1 in 4100 individuals in European (non-Finnish) populations and 1 in 3000 individuals in Middle Eastern populations (data from the gnomAD database). The patient is a veteran who initially presented to our clinic with a gradual decline in vision and pigmentary changes that started 40 years ago, with the right eye more affected than the left. The patient had bilateral cataract surgery.

During his most recent visit, his most recent BCVA was 20/70 in the right eye and 20/30 in the left eye. The FF revealed bilateral outer retinal atrophy sparing the central fovea, with a bull’s eye pattern surrounding the fovea (Figure 2C,D). Bone spicules were dispersed across the retina (360°), accompanied by pale optic discs, attenuated retinal vessels, and generalized macular atrophy. The GVF demonstrated an inferior residual crescent in both eyes and a small central visual field. The SD-OCT shows diffuse loss of the ellipsoid zone and outer nuclear layer, sparing part of the central fovea in the left eye.

### 3.2. VMD Patients

**Figure 3:**
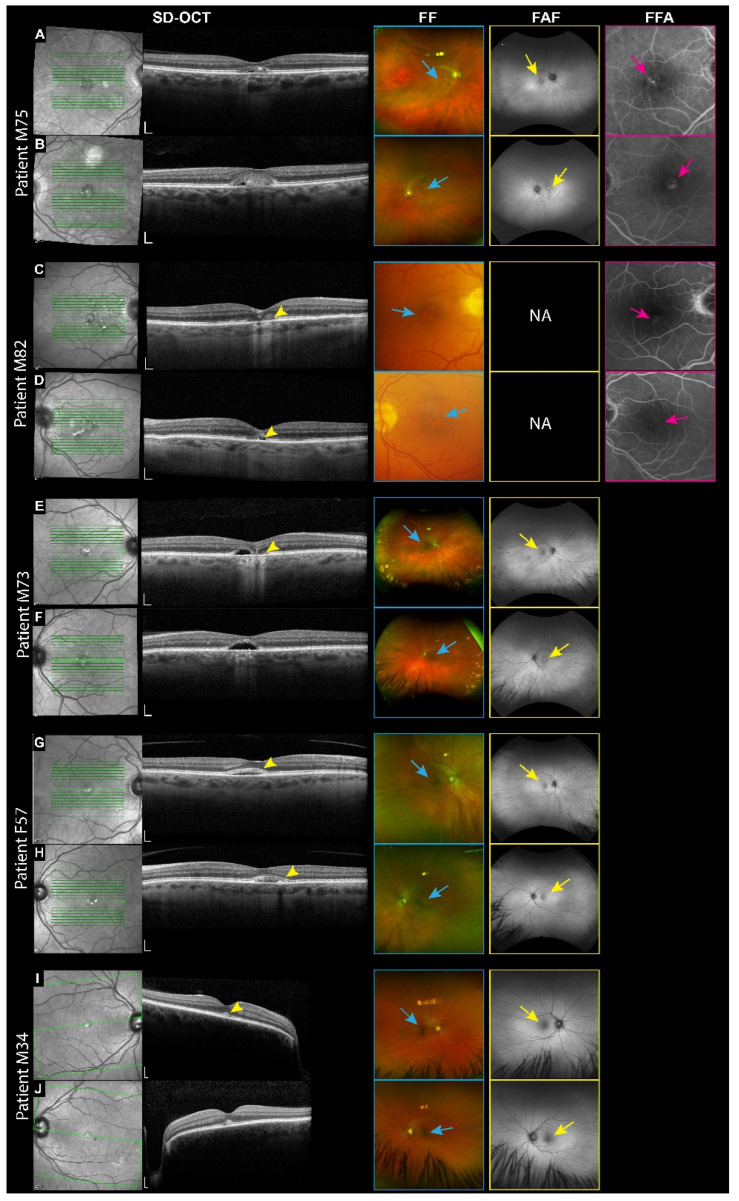
Multimodal imaging of patients with VMD associated with IMPG1 and IMPG2 mutations. Right eye top, left eye bottom for each patient. (**A**,**B**) Patient M75: SD-OCT reveals subretinal vitelliform material in the macula. FF shows hypopigmentation of the fovea (blue arrows). FAF shows hypo-autofluorescence in the right and left macula (yellow arrow). The FFA demonstrates early blockage and late window defects (Pink arrows). (**C**,**D**) Patient M82: SD-OCT displays foveal atrophy in the right eye and a vitelliform lesion with ellipsoid zone atrophy in the left eye (yellow arrowhead). The FF shows hypopigmentation of the fovea bilaterally (blue arrows). The FFA shows window defects (pink arrows). (**E**,**F**) Patient M73: SD-OCT reveals bilateral macular subretinal hyporeflective space and a reduced ellipsoid in the right eye (yellow arrowhead). The FF shows hypopigmentation of the fovea (blue arrows). The FAF shows bilateral hyper-autofluorescence (yellow arrows). (**G**,**H**) Patient F57: SD-OCT shows a vitelliform lesion in the macula of both eyes (yellow arrowheads). The FF and FAF reveal bilateral hypopigmentation and hypo-autofluorescence (blue and yellow arrows, respectively). (**I**,**J**) Patient M34: SD-OCT detects bilaterally vitelliform lesions in the fovea (yellow arrowheads). The FF and FAF reveal bilateral hypopigmentation and hypo-autofluorescence (blue and yellow arrows, respectively). NA: Not available.

Of the five patients diagnosed with VMD, only one, Patient M75, carries a mutation in the *IMPG1* gene. This 75-year-old male harbors a heterozygous missense mutation (p.Asp423Glu) located between the SEA1 and SEA2 domains. Subretinal vitelliform material was detected bilaterally in the macula by SD-OCT (Figure 3A,B). The FF shows hypopigmentation in the macula of both eyes. Hypo-autofluorescence, suggesting retinal atrophy, was observed in the right macula, while hyper-autofluorescence, indicative of vitelliform deposits, was noted in the left macula using FAF. Fluorescein angiography revealed early blockage and late-stage window defects, indicating RPE breakdown in both eyes. The patient had a superior macula nevus seen on the infrared and FF. His most recent BCVA was 20/40 in both eyes.

Among the patients with *IMPG2* mutations, Patient M82 is the only one with a missense mutation (p.Leu249Phe) affecting the SEA1 domain. This mutation highlights the potential significance of this domain for IMPG2 protein function. Patient M82 is an 82-year-old male diagnosed with late-stage VMD and foveal atrophy. SD-OCT of the right eye showed attenuated ellipsoid and retinal thinning, while the left eye displayed a vitelliform lesion with a small ellipsoid zone disruption. The FF shows hypopigmentation of the fovea in both eyes. The FFA demonstrates a bilateral window defect. His BCVA was 20/30 in the right eye and 20/25 in the left eye.

Patients M73 and F57 both have a heterozygous nonsense mutation (p.Arg964*) affecting the SEA2 domain of the *IMPG2* gene (Figure 1). These patients are unrelated. The Arg964* variant is a rare mutation that is most common in African and African American populations (1 in 18,700) and in European (non-Finnish) populations (1 in 59,000), according to gnomAD data. Patient M73, a 73-year-old male, presented with bilaterally macular subretinal hyporeflective spaces on SD-OCT, suggesting the reabsorption of vitelliform material (Figure 3E,F). Additional findings included ellipsoid disruption. The FF has bilateral fovea hypopigmentation, scattered dot-blot hemorrhages, and peripheral retinal “cobblestone” degeneration, likely attributable to the patient’s history of type II diabetes and advanced age. The FAF showed hyper-autofluorescence bilaterally, suggesting vitelliform deposits. His BCVA was 20/100 OD and 20/50 OS. Patient F57 is a 57-year-old female with a BCVA of 20/20 OU, exhibiting subretinal vitelliform lesions in the macula of both eyes on SD-OCT (Figure 3G,H). She was referred to the clinic for asymptomatic retinal lesions discovered by an unrelated retina screening. The FF shows hypopigmentation of the fovea in both eyes. FAF showed hypo-autofluorescence bilaterally, which was more pronounced in the left eye. 

Patient M34 is a 34-year-old male who carries a heterozygous nonsense mutation in *IMPG2* (p.Arg1088*), located within the EGF-like 2 domain, a variant previously described in the literature [22,24]. The patient was referred from the glaucoma clinic, where he was seen for juvenile glaucoma. The SD-OCT shows subretinal vitelliform lesions on both eyes. The FF is primarily indicative of glaucomatous optic nerves and mild macular hypopigmentation. FAF showed faint hyper-autofluorescence. His BCVA was 20/20 OU.

### 3.3. Pathogenicity Prediction

In silico prediction tools were used to evaluate the pathogenicity of variants in IMPG1 and IMPG2 proteins. For IMPG2, the variants p.Arg1088* (Stop gained) and p.Arg964* (Stop gained) are predicted to be pathogenic due to the premature stop codons that lead to truncated protein products. The missense variants p.Cys1019Tyr, p.Leu249Phe, and p.Arg131Cys are also classified as pathogenic, with SIFT, PolyPhen, and MutationTaster scoring them as likely damaging to protein function. In IMPG1, the novel variant p.Asp423Glu is predicted to be pathogenic, while the known variant p.Leu740Phe is considered likely benign. The details of these findings are shown in Table 2.

### 3.4. Prediction of Protein Stability for Point Mutations

We evaluated the impact of point mutations on the stability of the IMPG1 and IMPG2 proteins using several in silico tools, including mCSM, DUET, iMutant, and MuPRO. Utilizing multiple tools minimizes the risk of inaccurate predictions, ensuring more reliable results. The predicted free energy change (ΔΔG) values and their respective stability effects are summarized in Table 3. Our analysis revealed that all the variants, including p.Leu740Phe, p.Asp423Glu, p.Cys1019Tyr, p.Leu249Phe, and p.Arg131Cys, lead to destabilizing effects on the proteins, with the magnitude of destabilization varying across different tools. Specifically, the p.Leu249Phe variant exhibited the largest destabilizing effect, followed by the other variants. For the IMPG1 variants, both p.Leu740Phe and p.Asp423Glu are predicted to decrease protein stability. These results suggest that these point mutations may contribute to protein destabilization and potentially affect the function of the corresponding proteins.

### 3.5. Analysis of Interatomic Interactions in Reference and Mutant IMPG1/2 Proteins

**Figure 4:**
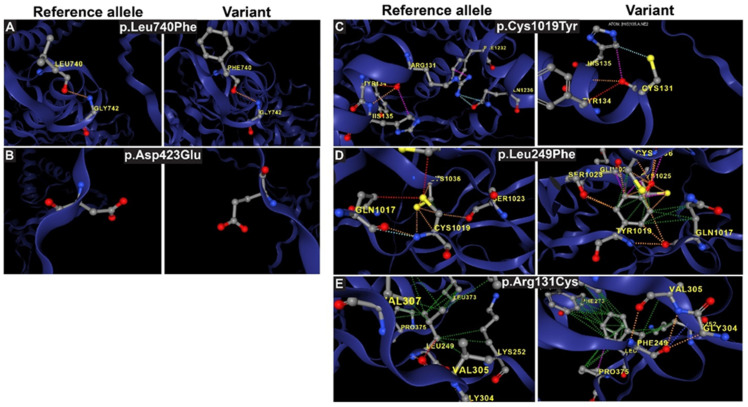
Molecular interactions and bond changes between reference and mutant forms of IMPG1 and IMPG2 are shown. The analysis evaluates the structural impact of each variant on protein stability and function. Variants p.Leu740Phe and p.Asp423Glu in IMPG1 are depicted in subpanels (**A**,**B**), respectively. Variants p.Cys1019Tyr, p.Leu249Phe, and p.Arg131Cys in IMPG2 are shown in subpanels (**C**–**E**). The left panel illustrates IMPG1 variants, while the right panel displays IMPG2 variants.

We analyzed the interatomic interactions within the reference and mutant forms of IMPG1 and IMPG2 to evaluate the structural consequences of specific point mutations (Figure 4). In IMPG1, no significant differences were observed in hydrogen bonds, van der Waals interactions or polar contacts between the reference and mutant proteins. This suggests that the p.Asp423Glu and p.Leu740Phe mutations (Figure 4A,B) do not disrupt the structural integrity or alter the interatomic interaction network of IMPG1. However, the functionality of the domain can be severely affected. In particular, the p.Leu740Phe mutation in the EGF-like domain adjacent to the autoproteolytic SEA2 domain is linked to RP (Figure 1).

However, physicochemical analysis revealed notable changes between the reference and mutant forms of IMPG1, indicating potential structural impacts. The molecular weight increased from 89.387 KDa in the reference protein to 89.419 KDa in the p.Asp423Glu mutant and 89.421 KDa in the p.Leu740Phe mutant, reflecting the incorporation of heavier residues. The p.Asp423Glu mutation was associated with a slightly higher theoretical isoelectric point, suggesting alterations in charge distribution (Table 4). Furthermore, the instability index increased in the p.Asp423Glu mutant, indicating reduced stability, while the aliphatic index decreased in the p.Leu740Phe mutant, suggesting diminished thermostability. These findings highlight potential structural and functional implications of the mutations, even in the absence of significant changes in the interatomic interaction network.

The *IMPG2* p.Cys1019Tyr mutation, located in the EGF-like domain, introduces significant changes to the interaction network. In the reference protein, Cysteine 1019 interacts with Serine 1023 through polar bonds and with Cysteine 1036 via hydrogen bonds (Figure 4C). In the mutant protein, Tyrosine 1019 replaces Cysteine 1019, disrupting these original interactions. Instead, Tyrosine 1019 forms new bonds with Serine 1023, Glutamine 1017, and Cysteine 1025. These alterations disturb the native interaction network that stabilizes the EGF-like domain, leading to further destabilization of the protein structure.

The p.Leu249Phe mutation further compromises the structural integrity of the SEA domain (Figure 4D). The reference protein, Leucine 249 (Leu249), forms hydrogen bonds with Valine 305 (Val305), Lysine 252 (Lys252), Leucine 373 (Leu373), and Valine 307 (Val307). The substitution of leucine with phenylalanine at position 249 disrupts these interactions. Additionally, changes to polar and hydrogen bonding networks involving Valine 306 (Val306), Glycine 304 (Gly304), and Phenylalanine 273 (Phe273) further destabilize the SEA domain, weakening its structural integrity.

In the reference IMPG2 protein, the side chain of Arginine 131 (Arg131) forms hydrogen bonds with Tyrosine 134 (Tyr134) and van der Waals interactions with Glutamine 1236 (Gln1236) (Figure 4E). However, the p.Arg131Cys mutation introduces a Cysteine at position 131, disrupting these critical interactions. Specifically, the hydrogen bond with Tyr134 and the van der Waals interaction with Histidine 135 (His135) are lost. This disruption destabilizes the protein structure, particularly within the highly conserved region spanning the signal sequence and the SEA1 domain, which is essential for maintaining structural and functional integrity. These analyses highlight how specific mutations in IMPG2 alter crucial interatomic interactions, destabilizing highly conserved domains.

## 4. Discussion

Inherited retinal diseases (IRDs) are a diverse group of conditions that collectively affect approximately 1 in 4000 individuals worldwide and are a leading cause of blindness in working-age adults [18,50]. While the prevalence varies depending on the specific type, RP is one of the most common forms [17].

The genes *IMPG1* and *IMPG2* have been observed to be two of many underlying genetic causes of RP and VMD. Depending on the variant and inheritance pattern, individuals affected can have variable presentations. In this study, we analyzed seven unrelated patients with either *IMPG1* or *IMPG2* gene mutations. From our original cohort of 480 IRD patients, seven met our study criteria, suggesting a prevalence of 1 in 68 for *IMPG1* or *IMPG2* related conditions, particularly within the American Appalachian population. To our knowledge, this is the first study to report the penetrance of *IMPG1* and *IMPG2* mutations in IRDs.

IMPG1 and IMPG2 proteoglycans undergo proteolytic cleavage at the carboxy-terminal SEA domain [16]. Patients developing RP linked to mutations in *IMPG1* have been reported to be dominant and exclusively affecting the proteolytic SEA domain of the protein [49]. Notably, patient F32 exhibited a heterozygous missense mutation in the EGF-like domain adjacent to the SEA domain, specifically affecting this region. This finding suggests that the EGF-like domain might play a role in the proper proteolysis of IMPG1.

Interestingly, the Leu740Phe variant associated with this mutation has a relatively high prevalence in the Caucasian/European (non-Finnish) population, occurring in approximately 1 in 332 individuals, according to gnomAD. This prevalence implies that the variant may also be present in healthy individuals. However, due to the lack of detailed phenotypic and age-related data for carriers, its penetrance and potential for age-related pathogenicity remain unclear. Further studies, including clinical evaluations of carriers and functional investigations, are essential to establish its pathogenicity and any age-related effects.

It is also important to note that this patient had two diseased gene variants: *IMPG1* and *JAG1*. *JAG1* mutations have been associated with Alagille syndrome, causing macular atrophy [51,52], and with autosomal dominant familial exudative vitreoretinopathy (FEVR) [53]. However, her symptoms did not align with those typically seen in Alagille or FEVR [54]. Despite our computational predictions that classified the IMPG1 p.Leu740Phe mutation as likely benign, the physicochemical analyses revealed notable changes, including increased molecular weight, higher theoretical isoelectric point, increased instability index, and decreased aliphatic index, suggesting reduced stability and thermostability. In addition, it is important to note that IMPG1 and IMPG2 proteins are extremely glycosylated and chondroitin sulfate proteoglycans that require several post-translational modifications [16]. Therefore, small changes in the highly conserved EGF-like domain may lead to significant changes in protein glycosylation or proteolysis.

Patient M83 is heterozygous at two locations: p.Cys1019Tyr and p.Arg131Cys, suggesting a recessive mode of inheritance. This type of compound heterozygous inheritance for RP had been previously reported by Habibi and colleagues [55]. Mutation p.Cys1019Tyr has been previously observed in patients with autosomal recessive RP [56], and p.Arg131Cys has been reported in ClinVar. The patient does have heterozygous variations in other genes with uncertain pathology significance (ALMS1, CEP164, COL11A1, CPLANE1, EYS, KIF7, NPHP4, and PHYH). It appears that the RP affects the inferior retina first, which may indicate a role of sunlight and exposure of the retina to light [57]. Both RP patients experienced visual field limitations in the superior visual field. The 83-year-old patient, despite his age, retained a central visual acuity of 20/30 and preserved a bilateral inferior visual field crescent. Cystoid maculopathy did not occur in either young or old patients. Based on our observations, IMPG1 and IMPG2 may be implicated in sector RP.

For VMD, most cases in the literature describe an autosomal dominant inheritance pattern [58,59]. All our VMD cases followed this inheritance pattern. Notably, patient M75 harbored a novel missense mutation, p.Asp423Glu, in IMPG1, marking its first reported association with VMD.

Patient M82 carried an *IMPG2* p.Leu249Phe variant, which significantly destabilized the non-proteolytic SEA domain, underscoring the importance of this domain for IMPG2 structural integrity. For Patient M73, along with an *IMPG2* variant, he also has a *BEST1* gene variant. Mutations in BEST1 are linked to Best-VMD. However, the patient’s EOG results were normal, a finding inconsistent with Best disease but more indicative of IMPG2-associated retinal dystrophy. Notably, this case was previously presented at the International Society for Clinical Electrophysiology of Vision (ISCEV) 2022 meeting [60].

Patient M34 was the youngest of the VMD patients at age 34. His BCVA remained at 20/20 bilaterally, and his vitelliform lesion was only detected through his SD-OCT performed at the time of the glaucoma visit. Interestingly, in most VMD patients, visual acuity was not significantly reduced. The only exception is patient M73, who has a BCVA of 20/100 OD and 20/50 OS.

Among patients harboring IMPG2 mutations, there were two unrelated individuals, patients M73 and F57, who both had VMD and had a heterozygous variant p.Arg964*. Both patients have similar vitelliform macular lesions. However, the oldest patient (M73) presents with peripheral cobblestone lesions, and the macular lesions have increased autofluorescence. Patient M34 carried the p.Arg1088* variant, which has been previously reported in the literature [22,24]. Notably, while this variant resulted in VMD in our patient, the patient studied by Van Huet [24] exhibited RP. A key genetic difference is that the patient in Van Huet’s study carried a homozygous mutation, whereas our patient, M34, carried a heterozygous mutation at the same genetic location. This distinction highlights how the inheritance pattern of the mutation may influence the clinical presentation.

Mutations in *IMPG1* and *IMPG2* were further investigated through an in-depth in silico analysis, focusing on protein stability, interatomic interactions, and the structural consequences of these variants. Pathogenicity predictions conducted through computational tools revealed that all mutations analyzed in this study were associated with significant structural and functional variations in the proteins.

This study supports the association of *IMPG1* and *IMPG2* mutations with retinal dystrophies and implies the prevalence of these mutations in IRDs in the Caucasian population for the first time. It also sheds light on the disease pathophysiology and paves the way for the development of targeted therapies. Consequently, animal models that replicate key aspects of the disease will enhance our understanding of the molecular mechanisms affecting the interphotoreceptor matrix and provide a platform to evaluate effective therapeutic strategies.

## Figures and Tables

**Table 1:** Clinical and genetic characteristics of patients with mutations in the IMPG1 and IMPG2 genes. The table summarizes patient sex and age, gene involved, the specific cDNA mutation, the corresponding protein change, inheritance patterns (Het = heterozygous, D = dominant, R = recessive), type of mutation (missense or nonsense), presentation of the associated retinal disease (RP = retinitis pigmentosa, VMD = vitelliform macular dystrophy), and the best corrected visual acuity (VA) in each eye (OD = right eye, OS = left eye).

Patient ID#: Sex and Age (Y)	Gene Involved	cDNA	Protein	Zygosity and Inheritance	Type of Mutation	Presentation of Disease	OD–OS VA
F32	*IMGP1*	c.2218C>T	p.Leu740Phe	Het; D	Missense	Sectoral RP	20/25–20/25
M83	*IMPG2*	c.3056G>A & c.391C>T	p.Cys1019Tyr & p.Arg131Cys	Het for both genes; R	Missense	RP	20/70–20/30
M75	*IMPG1*	c.1269C>A	p.Asp423Glu	Het; D	Missense	VMD	20/40–20/40
M82	*IMPG2*	c.745C>T	p.Leu249Phe	Het; D	Missense	VMD	20/30–20/25
M73	*IMPG2*	c.2890C>T	p.Arg964*	Het; D	Nonsense	VMD	20/100–20/50
F57	*IMPG2*	c.2890C>T	p.Arg964*	Het; D	Nonsense	VMD	20/20–20/20
M34	*IMPG2*	c.3262C>T	p.Arg1088*	Het; D	Nonsense	VMD	20/20–20/20

**Table 2:** Table displaying predicted functional impact scores for *IMPG2* and *IMPG1* gene variants. SIFT and PolyPhen scores indicate the potential impact of amino acid changes, while CADD and FATHMM scores assess variant deleteriousness.

Gene	Variant	SIFT Score	PolyPhen Score	CADD Score	FATHMM Coding	MutationTaster Score	Prediction	Existing Variant ID (ClinVar)
*IMPG1*	p.Leu740Phe	0.09	0.575	3.6	-	22	Likely Benign	rs15048636
*IMPG1*	p.Asp423Glu	0.02	0.98	2.5	-	45	Pathogenic	Novel
*IMPG2*	p.Cys1019Tyr	0	0.996	25.8	0.0376	46	Pathogenic	rs2107208112
*IMPG2*	p.Arg131Cys	0	0.645	21.7	0.0577	91	Pathogenic	rs150344327
*IMPG2*	p.Leu249Phe	0	0.998	22.6	0.0076	78	Pathogenic	rs376443291
*IMPG2*	p.Arg964*	-	-	11.2	0.011	58	Pathogenic	rs267606975
*IMPG2*	p.Arg1088*	-	-	13.4	0.0513	29	Pathogenic	rs199867882

**Table 3:** Predicted stability changes (ΔΔG) for IMPG1 and IMPG2 variants. Predictions indicate destabilizing (DSTB) or decreased stability (DecStab) effects on protein stability according to mCSM, SDM, DUET, iMutant, and MuPRO web resources.

Variant	Gene	mCSM AAG	SDM AAG	DUET AAG	iMutant AAG	MuProAAG
p.Leu740Phe	*IMPG1*	−0.588 (DSTB)	−0.33 (DSTB)	−0.591 (DSTB)	−0.85 (DSTB)	−0.930 (DecStab)
p.Asp423Glu	*IMPG1*	−0.653 (DSTB)	−0.52 (DSTB)	−0.138 (DSTB)	−0.76 (DecStab)	−0.478 (DecStab)
p.Cys1019Tyr	*IMPG2*	−0.826 (DSTB)	−0.78 (DSTB)	−0.85 (DSTB)	−0.14 (DSTB)	−1.266 (DSTB)
p.Arg131Cys	*IMPG2*	−0.007 (DSTB)	−0.22 (DSTB)	−0.018 (DSTB)	−1.13 (DSTB)	−0.627 (DSTB)
p.Leu249Phe	*IMPG2*	−1.743 (DSTB)	−0.66 (DSTB)	−1.77 (DSTB)	−0.55 (DSTB)	−1.525 (DSTB)

**Table 4:** Physicochemical properties of reference and mutant IMPG1 proteins. This table summarizes the molecular weight, theoretical isoelectric point (pI), instability index, aliphatic index, and grand average of hydropathicity (GRAVY) for the reference IMPG1 protein and its two mutant variants (p.Asp423Glu and p.Leu740Phe).

Physiochemical Properties	Reference Allele	p.Asp423Glu	p.Leu740Phe
Molecular weight	89,387	89,419	89,421
Theoretical pI	4.79	4.81	4.79
Total number of atoms	12,450	12,457	12,451
Instability Index	53.57	53.76	53.16
Aliphatic Index	75.63	75.63	75.14
GRAVY	−0.482	−0.474	−0.483

## Data Availability

No new data were created or analyzed in this study. Therefore, no data sharing repository is provided. This research involved human subjects, and due to ethical and privacy considerations, sharing patient-related data is restricted in compliance with applicable regulations. For further details, please refer to the MDPI Research Data Policies at https://www.mdpi.com/ethics.
